# Tailoring the Interfacial Band Offset by the Molecular Dipole Orientation for a Molecular Heterojunction Selector

**DOI:** 10.1002/advs.202101390

**Published:** 2021-09-09

**Authors:** Jung Sun Eo, Jaeho Shin, Seunghoon Yang, Takgyeong Jeon, Jaeho Lee, Sanghyeon Choi, Chul‐Ho Lee, Gunuk Wang

**Affiliations:** ^1^ KU‐KIST Graduate School of Converging Science and Technology Korea University 145, Anam‐ro, Seongbuk‐gu Seoul 02841 Republic of Korea; ^2^ Department of Integrative Energy Engineering Korea University 145, Anam‐ro, Seongbuk‐gu Seoul 02841 Republic of Korea

**Keywords:** 2D semiconductor, molecular dipole moment, molecular electronics, molecular heterojunction, molecular selector

## Abstract

Understanding and designing interfacial band alignment in a molecular heterojunction provides a foundation for realizing its desirable electronic functionality. In this study, a tailored molecular heterojunction selector is implemented by controlling its interfacial band offset between the molecular self‐assembled monolayer with opposite dipole orientations and the 2D semiconductor (1_L_‐MoS_2_ or 1_L_‐WSe_2_). The molecular dipole moment direction determines the direction of the band bending of the 2D semiconductors, affecting the dominant transport pathways upon voltage application. Notably, in the molecular heterostructure with 1_L_‐WSe_2_, the opposite rectification direction is observed depending on the molecular dipole moment direction, which does not hold for the case with 1_L_‐MoS_2_. In addition, the nonlinearity of the molecular heterojunction selector can be significantly affected by the molecular dipole moment direction, type of 2D semiconductor, and metal work function. According to the choice of these heterojunction constituents, the nonlinearity is widely tuned from 1.0 × 10^1^ to 3.6 × 10^4^ for the read voltage scheme and from 0.4 × 10^1^ to 2.0 × 10^5^ for the half‐read voltage scheme, which can be scaled up to an ≈482 Gbit crossbar array.

## Graphical abstract

By tailoring the interfacial band offset with the molecular dipole orientation, molecular heterojunction selector is presented. Based on diverse interfacial band offsets achieved by changing the heterojunction constituents, such as the molecular dipole moment direction, types of two‐dimensional transition metal dichalcogenide, and work function of the electrodes, the nonlinearity is widely tuned, scaling up to an ≈482 Gbit crossbar array.

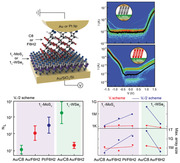

## 1 Introduction

Implementing a functional molecular‐scale device is an essential step toward realizing the ultimate electronic device scaling beyond the complementary metal‐oxide‐semiconductor scaling limit without increasing the thermal budget.^[^ ^1^ ^]^ Generally, the molecular building block placed between conductive electrodes governs the electronic functionalities because the molecular structure and its corresponding discrete orbital states can determine the dominant charge transport behaviors.^[^ ^2^, ^3^, ^4^, ^5^, ^6^, ^7^, ^8^, ^9^ ^]^ Therefore, using a specific designed molecular species is a general and rational way to realize a functional molecular device. For example, molecular self‐assembled monolayers (SAMs) composed of aliphatic groups (such as n‐alkanethiol molecules) only act as simple insulators.^[^ ^2^, ^3^ ^]^ In contrast, molecules with an aliphatic backbone but an altered anchoring group of electroactive moieties (e.g., fullerene and ferrocene) can rectify the current.^[^ ^9^, ^10^, ^11^ ^]^ In addition, diarylethene and azobenzene molecules can exhibit photoswitching behavior depending on the wavelength of light.^[^ ^12^, ^13^ ^]^ Molecular resistive random‐access memory was also reported based on alkyl molecular SAMs containing functional MV^2+^ (where MV is methylviologen) redox units, which can adjust the molecular energy level via the various oxidation states.^[^ ^14^ ^]^

In contrast to this conventional strategy, which uses the functionality of the molecule itself, by exploiting a combination of molecules and various nanomaterials, a variety of functional molecular heterojunctions have been recently developed and suggested.^[^ ^15^, ^16^, ^17^, ^18^, ^19^, ^20^ ^]^ For example, Vezzoli et al. demonstrated enhancement of the photocurrent in a metal‐molecule‐semiconductor nanodevice by adjusting the doping density of GaAs and the frontier molecular orbital levels.^[^ ^16^ ^]^ They insisted that the interfacial energy gap between the molecular energy level and the Fermi level (*E*_F_) of the metal is a significant factor determining the magnitude of the photocurrent. Margapoti et al. reported an abrupt change in the electrical characteristics of an Au/photochromic azobenzene molecular SAM/single‐layer MoS_2_ junction depending on the *trans* and *cis* molecular configuration modulated by light.^[^ ^17^ ^]^ Lastly, we reported molecular heterojunctions with nonfunctional molecules and different types of 2D transition metal dichalcogenide (TMD) semiconductors (MoS_2_ and WSe_2_), which exhibit different transport pathways upon the application of different voltage polarities, realizing a molecular‐scale diode.^[^ ^18^ ^]^ These methods can all provide a novel strategy for designing functional molecular junctions distinguished from the traditional forms in which a specifically designed molecular system has been an absolute necessity to provide electronic functionality.

Apart from the functionalities of molecular building blocks, the molecular dipole moment can also be considered an essential variable determining the charge transport. It can adjust the direction of the interfacial band bending and local electric field induced in the junction. In general, the magnitude of the molecular dipole moment of the SAM attached to the metal substrate can determine the degree of change in its work function by shifting the relative vacuum levels.^[^ ^19^, ^20^, ^21^, ^22^, ^23^, ^24^, ^25^, ^26^ ^]^ In addition, orientation of the molecular dipole moment along an external electric field can significantly affect the transport conduction through the molecular junction. For example, Baghbanzadeh et al. designed a specific molecular SAM with functional dipolar groups, exhibiting a rectification feature up to ≈20.^[^ ^27^ ^]^ Kovalchuk et al. ascribed the dipole‐induced asymmetric tunneling conduction in eutectic Ga‐In/pyrimidyl‐based molecules/Au.^[^ ^28^ ^]^ Moreover, many studies on organic thin‐film transistors (OTFTs) with various molecular dipole moments have also been performed.^[^ ^23^, ^24^ ^]^ Molecular dipoles can create an electrostatic potential that withdraws or injects charge carriers from/into the semiconductor channel according to the molecular dipole moment direction. This leads to a change in the threshold and the turn‐on voltage of the OTFT device. Despite this crucial role of the molecular dipole moment in the electronic transport in junctions, molecular heterojunctions with different molecular dipoles and orientations have rarely been studied. In addition, a comprehensive understanding of how the charge transport can be affected by the direction of the molecular dipole moment in the molecular heterojunction and what electronic functionality can be designed and applied is not yet systematically established.

In this study, we investigate the charge transport according to the interfacial band offsets in various molecular heterojunctions consisting of molecular SAMs with opposite dipole orientations and different types of monolayer‐thick 2D TMD (1_L_‐MoS_2_ or 1_L_‐WSe_2_) based on a conducting atomic force microscopy (CAFM) technique. According to the choice of constituents in the molecular heterojunction, such as the direction of the molecular dipole moment, the type of 2D TMD, and the metal work function, diverse interfacial band offsets can be established, tuning the rectification features and direction. Notably, this interfacial band engineering can also be used to widely adjust the degree of current increase and the nonlinearity of the rectification characteristics upon voltage application. This characteristic suggests the potential for a tailored molecular‐scale heterojunction selector that can prevent sneak current flow in a crossbar array structure. Based on the readout margin estimated by conventional one bit‐line pull‐up simulation for different reading voltage schemes, a switching array integrated with this molecular selector could be scaled up to ≈482 Gbit. This result could pave a novel way to extend the application region of the molecular junction.

## 2 Results and Discussion

**Figure 1:**
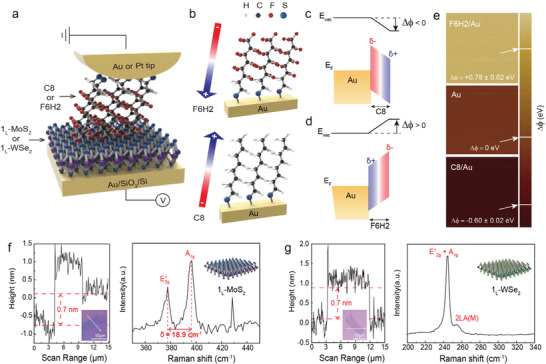
Molecular heterojunctions with opposite orientations of the molecular dipole moment. a) Schematic of the molecular heterojunction consisting of Au (or Pt)‐tip/molecule/1_L_‐2D TMD (1_L_‐MoS_2_ and 1_L_‐WSe_2_) on a Au/SiO_2_/Si substrate, which was investigated by the CAFM technique with *F*_L_ = 1 nN. b) Illustration of the SAM‐modified Au metal substrate with F6H2 (top) and C8 (bottom). The arrow indicates the orientation of the molecular dipole moment. c,d) Schematics of energy level diagrams for the F6H2 and C8 SAMs on Au metal substrates. *δ* ^+^ and *δ* ^−^ indicate the positive and negative charge distribution of the molecular SAMs on Au, whose direction determines the sign of Δ*ϕ*, that is, Δ*ϕ* > 0 for F6H2 and Δ*ϕ* < 0 for C8 SAMs. e) Surface potential images for F6H2/Au (top), bare Au (middle), and C8/Au (bottom) probed by SKPM. Topological line profiles and corresponding optical images of 1_L_‐2D TMD/SiO_2_/Si with an arrow indicating the investigation range of the line‐profiles and Raman spectra for f) 1_L_‐MoS_2_ and g) 1_L_‐WSe_2_.

**Figure** **1**a shows a schematic of the molecular heterojunction structure composed of a molecular SAM and a 2D TMD semiconductor (1_L_‐MoS_2_ or 1_L_‐WSe_2_) stacked on an Au/SiO_2_/Si substrate, for which the CAFM technique is used to investigate the electrical properties. Two different molecular species are selected: 1‐octanethiol (CH_3_(CH_2_)_7_SH, denoted C8) and tridecafluoro‐1‐octanethiol (CF_3_(CF_2_)_5_(CH_2_)_2_SH, denoted F6H2) have opposite charge distributions driven by the different terminal/backbone groups of —CH*_n_* and —CF*_n_*, exhibiting opposite dipole moment directions with respect to the Au metal (Figure 1b).^[^ ^19^, ^29^, ^30^ ^]^ The dipole moment direction is defined by the direction from the negative charge to the positive charge for the molecule. Because of the relatively high electronegativity of the F atoms for F6H2, the dipole moment of F6H2 is oriented from the molecular terminal (—CF_3_) to the Au metal.^[^ ^22^, ^26^ ^]^ In contrast, the dipole moment of C8 is oriented from the Au metal to the molecular terminal (—CH_3_). As shown in Figure 1c,d, the molecular SAMs covalently bonded to the Au metal can shift the vacuum level (*E*_vac_) by the interfacial potential drop (Δ*ϕ*), changing the work function of the underlying Au metal.^[^ ^19^, ^30^, ^31^, ^32^ ^]^ The dipole moment direction determines the sign of Δ*ϕ* with respect to the Fermi level of the Au metal, enabling an increase or a decrease in the work function. The F6H2 molecules are expected to shift *E*_vac_ up (i.e., Δ*ϕ* > 0), while the C8 molecules on Au are expected to shift *E*_vac_ down (i.e., Δ*ϕ* < 0). To confirm the change in Δ*ϕ* with the molecular dipole moment direction, we performed scanning Kelvin probe microscopy (SKPM) on F6H2/Au, bare Au, and C8/Au substrates. SAMs of F6H2 have a positive Δ*ϕ* of 0.78 ± 0.02 eV, while SAMs of C8 have a negative Δ*ϕ* of −0.60 ± 0.02 eV, which are both in good agreement with the theoretical expectations and previous studies for similar molecular species, as shown in Figure 1e.^[^ ^19^, ^26^, ^29^ ^]^

Two different types of 2D TMD monolayers (n‐type (MoS_2_) and p‐type (WSe_2_)) on Au/SiO_2_/Si substrates were prepared by using the typical mechanical exfoliation and transfer method (see Experimental Section). The prepared MoS_2_ and WSe_2_ and their layer numbers were investigated by Raman spectroscopy, photoluminescence (PL) spectroscopy, and topological line profiling using AFM (Figure 1f,g; Figure S1, Supporting Information). The insets of Figure 1f,g show the optical images for TMDs, exhibiting that both are monolayer thick (1_L_, ≈0.7 nm^[^ ^33^ ^]^) based on the scanned topological profile. In the Raman spectra, both TMDs exhibit vibration modes corresponding to monolayer‐thick MoS_2_ and WSe_2_ (denoted 1_L_‐MoS_2_ and 1_L_‐WSe_2_).^[^ ^34^, ^35^ ^]^ The spacing between *E* ^1^_2g_ = 377 cm^−1^ and *A*_1g_ = 396 cm^−1^ is *δ* = 18.9 cm^−1^ for 1_L_‐MoS_2_, and overlapped spectra of *E* ^1^_2g_ and *A*_1g_ at 242 cm^−1^ are observed for 1_L_‐WSe_2_. After the dense formation of SAMs of both C8 and F6H2 on Au (or Pt) tips, each tip brought to the 2D TMD with the loading force *F*_L_ = 1 nN, and five different types of molecular heterojunctions were made: 1) Au‐tip/C8/1_L_‐MoS_2_/Au, 2) Au‐tip/F6H2/1_L_‐MoS_2_/Au, 3) Au‐tip/C8/1_L_‐WSe_2_/Au, 4) Au‐tip/F6H2/1_L_‐WSe_2_/Au, and 5) Pt‐tip/F6H2/1_L_‐MoS_2_/Au. For the electrical measurement, a voltage was applied to the bottom Au electrode, and the metal tip was grounded. Note that the change of molecular effective dipole moments according to contact curvature of the tip can be negligible. The details of the molecular effective dipole moment according to the contact curvature are described in Note S1, Figures S2–S4, Table S1, Supporting Information. The details of the sample preparation and electrical characterizations are described in the Experimental section.

**Figure 2:**
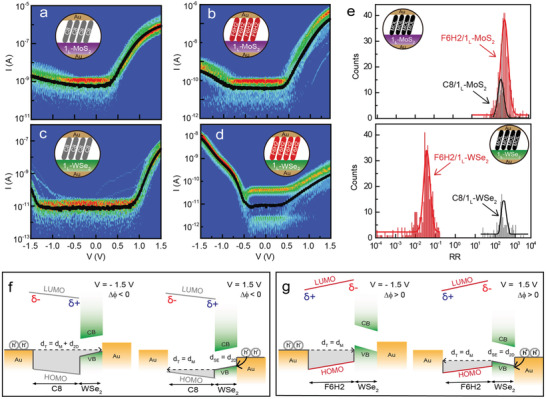
Rectification features and energy band offsets for the molecular heterojunctions. Statistical heat maps of *I−V* characteristics illustrated in heat maps for the molecular heterojunctions of a) Au‐tip/C8/1_L_‐MoS_2_/Au, b) Au‐tip/F6H2/1_L_‐MoS_2_/Au, c) Au‐tip/C8/1_L_‐WSe_2_/Au, and d) Au‐tip/F6H2/1_L_‐WSe_2_/Au. e) Statistical histogram of *RR* for the different molecular species (C8 in gray and F6H2 in red) on 1_L_‐MoS_2_ (top) and 1_L_‐WSe_2_ (bottom) junctions. The line curves are fitting results from Gaussian functions. Schematics of interfacial energy band offsets for the molecular heterojunctions with 1_L_‐WSe_2_, in which the rectification direction is opposite for the f) C8 and g) F6H2 molecules when *V* = −1.5 V (left) and *V* = 1.5 V (right) is applied.

**Figure** **2**a–d shows statistical heat maps of current‐voltage (*I–V*) characteristics for four different molecular heterojunctions: a) Au‐tip/C8/1_L_‐MoS_2_/Au, b) Au‐tip/F6H2/1_L_‐MoS_2_/Au, c) Au‐tip/C8/1_L_‐WSe_2_/Au, and d) Au‐tip/F6H2/1_L_‐WSe_2_/Au. Each *I–V* characteristic was measured at several positions (≈20 positions) in different junction samples. The black lines in Figure 2a–d exhibit the average *I–V* characteristics for the four different molecular heterojunctions. All *I–V* curves exhibit reproducible and reliable rectification behavior with a certain rectification ratio (*RR*), defined as *RR* = |*I* (*V* = 1.5 V)/*I* (*V* = −1.5 V)|. With this definition, if *RR* > 1, then the rectification direction is positive, while if 0 < *RR* < 1, then it is negative. Note that for the junctions composed of only the molecules junctions or only the 2D TMDs vertical junctions, the *I–V* characteristics exhibit a symmetric‐like behavior due to the single transport barrier between two Au electrodes (Figure S5, Supporting Information). As shown in Figure 2a,b, the statistical *RR*s for the C8/1_L_‐MoS_2_ and F6H2/1_L_‐MoS_2_ junctions are (4.2 ± 0.9) × 10^2^ and (5.9 ± 0.7) × 10^2^, respectively. Both junctions have positive rectification directions regardless of the molecular dipole direction (the top of Figure 2e). Note that except for the molecular dipole, C8 and F6H2 molecules are known to have a similar energy gap between the highest‐occupied molecular orbital (HOMO) and the lowest‐unoccupied molecular orbital (LUMO),^[^ ^36^ ^]^ and they are also similar in molecular length (≈1.2 nm).^[^ ^37^, ^38^, ^39^ ^]^ This rectification characteristic can be understood based on the different transport conductions driven by the change in interfacial energy band alignments depending on the voltage polarity.^[^ ^18^ ^]^ In this type of molecular heterostructure with 1_L_‐MoS_2_ (i.e., the majority carrier is an electron in this case), for example, the energy band alignment of the molecular SAM and 1_L_‐MoS_2_ can be independently varied according to the applied voltage polarity due to the different coupling strengths at the Au‐tip/molecule, molecule/1_L_‐MoS_2_, and 1_L_‐MoS_2_/Au interfaces.^[^ ^18^ ^]^ This interfacial band shift can lead to different transport pathways according to the voltage polarity, causing positive rectification characteristics (Figure S6, Supporting Information). Since both heterojunctions exhibit similar rectification characteristics, the molecular dipole moment direction does not seem to mainly affect the charge transport pathways.

However, in the type of molecular heterostructure with 1_L_‐WSe_2_ (i.e., the majority carrier is a hole in this case (Figure S7, Supporting Information)), interestingly, the rectification direction is opposite depending on the molecular dipole moment direction (Figure 2c–e). For example, the statistical *RR*s of C8/1_L_‐WSe_2_ and F6H2/1_L_‐WSe_2_ junctions are (3.3 ± 0.8) × 10^2^ and (2.2 ± 0.9) × 10^−2^, respectively, indicating opposite rectification directions. To systematically understand this opposite direction, the interfacial band alignments for both molecular heterojunctions are established according to the voltage polarities (at *V* = ± 1.5 V), as shown in Figure 2f,g. The molecular SAMs are chemically linked to the top Au tip by Au‐S covalent bonding for both energy bands. Therefore, the molecular HOMO and LUMO levels can follow the shift of *E*_F_ of the Au tip upon voltage application. However, the *E*_F_ of the bottom Au electrode can be strongly pinned at the mid‐gap state between the energy levels (conduction band (CB) and valence band (VB)) of 1_L_‐WSe_2_ due to the existence of vacancy states in the mechanically transferred TMD.^[^ ^40^, ^41^ ^]^ Therefore, the energy band of 1_L_‐WSe_2_ can shift along with the *E*_F_ of the bottom Au electrode according to the applied voltage. In the interfacial energy band alignment schematics, the effect of the molecular dipole moments can be additionally considered. Depending on the sign of Δ*ϕ* determined by the molecular dipole moment direction, the energy band edges of C8 and F6H2 are shifted downward or upward, causing the band bending of 1_L‐_WSe_2_ at the interface to differ (Figure 2f,g). For the C8/1_L_‐WSe_2_ junction (Δ*ϕ* < 0), two different transport pathways may be attributed according to *V* = ± 1.5 V, where one is the tunneling across both the C8 and 1_L_‐WSe_2_ barriers (*d*_T_ = *d*_M_ + *d*_2D_) at *V* = −1.5 V, and the other is the combination transport of Schottky emission (*d*_SE_ = *d*_2D_) and the tunneling (*d*_T_ = *d*_M_) across the 1_L_‐WSe_2_ and C8 barriers at *V* = 1.5 V (marked in gray in Figure 2f). Specifically, when *V* = −1.5 V, the charges (i.e., h^+^) transfer across *d*_T_ (= *d*_M_ *+ d*_2D_) from the top Au tip to the bottom Au electrode. During the charge transfer, the C8 barrier height (defined as the difference between the *E*_F_ of the top Au tip and the HOMO of C8 in this case) remains constant, while the effective tunneling barrier across 1_L_‐WSe_2_ is further reduced for a higher negative voltage. In contrast, when *V* = 1.5 V, the charge transfer direction is the opposite (i.e., from the bottom Au to the Au tip electrode), and the pathway is changed. Under this circumstance, the effective Schottky barrier at 1_L_‐WSe_2_/Au remains constant due to the Fermi level pinning effect, while the C8 barrier is further reduced for a higher positive voltage. Therefore, the effective C8 barrier height changes depending on the applied voltage polarity, which might be a major factor determining the asymmetricity of the transport current. In other words, the relatively low C8 tunneling barrier at *V* = 1.5 V results in a higher transport current than that at *V* = −1.5 V. In contrast, for the F6H2/1_L_‐WSe_2_ junction, the positive Δ*ϕ* due to the F6H2 SAMs on Au induces the opposite band bending (upward) of 1_L_‐WSe_2_, changing the major transport pathways. Charges mainly transfer across F6H2 (i.e., *d*_T_ = *d*_M_) only when *V* = −1.5 V with a high possibility (marked in gray in the left schematic of Figure 2g). This is because the VB edge of 1_L_‐WSe_2_ is located in the applied bias window due to the upward VB bending, enabling charge injection through the below VB. In contrast, when *V* = 1.5 V, the charges transfer through both Schottky emission (*d*_SE_ = *d*_2D_) and tunneling (*d*_T_ = *d*_M_) transport across the 1_L_‐WSe_2_ and F6H2 barriers (marked in gray on the right of Figure 2g), leading to a longer transport pathway than when *V* = −1.5 V. This difference results in the opposite rectification direction (a higher current at *V* = −1.5 V).

**Figure 3:**
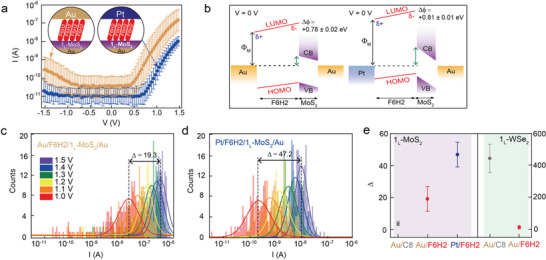
Comparison of rectification characteristics between Au‐tip/F6H2/1_L_‐MoS_2_/Au and Pt‐tip/F6H2/1_L_‐MoS_2_/Au junctions. a) Statistical *I−V* characteristics for the Au‐tip/F6H2/1_L_‐MoS_2_/Au (gold) and Pt‐tip/F6H2/1_L_‐MoS_2_/Au (blue) junctions. b) Schematics of interfacial energy band offsets for the Au‐tip/F6H2/1_L_‐MoS_2_/Au and Pt‐tip/F6H2/1_L_‐MoS_2_/Au junctions at the equilibrium condition with *V* = 0 V. The Pt tip increases *Φ*_M_ and induces a larger upward bending of the band of 1_L_‐MoS_2_ (green arrow) compared to the Au tip. Both junctions exhibit similar and positive values for Δ*ϕ*. Statistical histograms of the transport current upon applying voltages from 1.0 to 1.5 V for the c) Au‐tip/F6H2/1_L_‐MoS_2_/Au and d) Pt‐tip/F6H2/1_L_‐MoS_2_/Au junctions. e) *Δ* as a function of the molecular species (C8 and F6H2), 2D TMD (1_L_‐MoS_2_ and 1_L_‐WSe_2_), and top metal electrodes (Au and Pt tip).

To investigate the effect of the metal work function on the interfacial band alignment in the molecular heterojunction, we changed the top Au tip to the Pt tip. **Figure** **3**a shows the *I–V* characteristics for Au‐tip/F6H2/1_L_‐MoS_2_/Au and Pt‐tip/F6H2/1_L_‐MoS_2_/Au junctions. Compared with the Au‐tip/F6H2/1_L_‐MoS_2_/Au junction, the Pt‐tip/F6H2/1_L_‐MoS_2_/Au junction shows a slightly higher *RR* and a lower transport current (**Table** **1**, Figure S8, Supporting Information). Changing the metal work function in the molecular heterojunction is expected to bring a change in the interfacial band offset in terms of the molecular barrier and the degree of band bending due to the alleviation of the pinning effect^[^ ^42^ ^]^ (Figure 3b). A higher work function induced by the top Pt electrode can decrease the transport current because it creates a higher molecular barrier (*Φ*_M_, the difference between the *E*_F_ of top Pt and the LUMO of F6H2 in this case; see Figure S8, Supporting Information). This results in a larger band bending (upward) of 1_L_‐MoS_2_ compared to the use of the top Au electrode, as shown by the green arrows in Figure 3b. Note that F6H2 SAMs on Pt metal have a positive Δ*ϕ*, 0.81 ± 0.01 eV, measured by the SKPM technique (Figure S9, Supporting Information), similar to the F6H2 SAMs on Au. Another noticeable difference between the two molecular heterojunctions is that the degrees of the current increase under an applied positive voltage are different. Figure 3c,d shows the histograms of the transport currents for Au‐tip/F6H2/1_L_‐MoS_2_/Au and Pt‐tip/F6H2/1_L_‐MoS_2_/Au junctions as a function of the applied voltage (from 1.0 to 1.5 V), respectively. Note that the degree of the current increase is defined as *Δ* = (*I* (*V* = 1.5 V) − *I* (*V* = 1.0 V))/*I* (*V* = 1.0 V). The *Δ* for the Pt‐tip/F6H2/1_L_‐MoS_2_/Au junction is ≈47.2, more than twice the *Δ* for the Au‐tip/F6H2/1_L_‐MoS_2_/Au junction (≈19.3). We speculate that the *Δ* difference is associated with the upward degree (the green arrows) of the band bending of 1_L_‐MoS_2_ depending on the work function of the top metal electrode (Au and Pt) (Figure 3b). A larger upward bending of the CB in the Pt‐tip/F6H2/1_L_‐MoS_2_/Au junction can largely decrease the effective transport barrier determined by the F6H2 and 1_L_‐MoS_2_ barrier as the positive applied voltage is increased. This results in a relatively high *Δ* for the Pt‐tip/F6H2/1_L_‐MoS_2_/Au junction. Consequently, since the band bending of the 2D TMD is dependent on the molecular dipole moment, 2D TMD type, and metal work function, the *Δ* upon voltage application can be controlled according to these constituents of the molecular heterojunction (Figure 3e; Figure S10, Supporting Information). As shown in Figure 3e, in the molecular heterojunction with 1_L_‐MoS_2_, *Δ* increased by more than ten times (from 4.0 ± 1.0 to 47 ± 8) when the molecular species was changed from C8 to F6H2 and the top electrode was changed from Au to Pt metal. However, in the molecular heterojunction with 1_L_‐WSe_2_, *Δ* decreased by more than a few tens of times (from (4.4 ± 0.9) × 10^2^ to (1.2 ± 0.3) × 10^1^) when the molecular species was changed from C8 to F6H2. This opposite behavior of *Δ* is associated with the change in the type of 2D TMD (change in the majority carrier) that can affect the dominant transport pathway. Notably, *Δ* is strongly related to the nonlinearity (*N*_L_) of the rectification characteristics;^[^ ^43^ ^]^ hence, it could offer a distinctive design rule that can be suggested as a molecular selector.

**Table 1:** Summary of molecular heterojunction selectors

Electrode	2D types	Molecular species	*RR*	*V*_r_ scheme			*V*_r_ */2* scheme		
				*N*_L_	Avg. size	Max. size	*N*_L_	Avg. size	Max. size
Au/Au	1_L_‐MoS_2_	C8	(4.2 ± 0.9) × 10^2^	(3.7 ± 0.6) × 10^2^	0.9 K	2 M	(1.3 ± 0.3) × 10^1^	1.8 K	23 K
Au/Au	1_L_‐MoS_2_	F6H2	(5.9 ± 0.7) × 10^2^	(5.6 ± 0.7) × 10^2^	2.4 K	118 M	(1.1 ± 0.2) × 10^2^	0.1 M	439 M
Pt/Au	1_L_‐MoS_2_	F6H2	(6.2 ± 1.2) × 10^2^	(5.6 ± 1.0) × 10^2^	1.2 K	19 M	(3.7 ± 0.7) × 10^2^	1 M	9 G
Au/Au	1_L_‐WSe_2_	C8	(3.3 ± 0.8) × 10^2^	(2.3 ± 0.5) × 10^2^	1.8 K	5 M	(2.1 ± 0.4) × 10^3^	50 M	482 G
Au/Au	1_L_‐WSe_2_	F6H2	(2.3 ± 0.9) × 10^−2^	(4.0 ± 1.5) × 10^1^	0.6 K	889 K	(2.3 ± 0.3) × 10^1^	6 K	59 K

**Figure 4:**
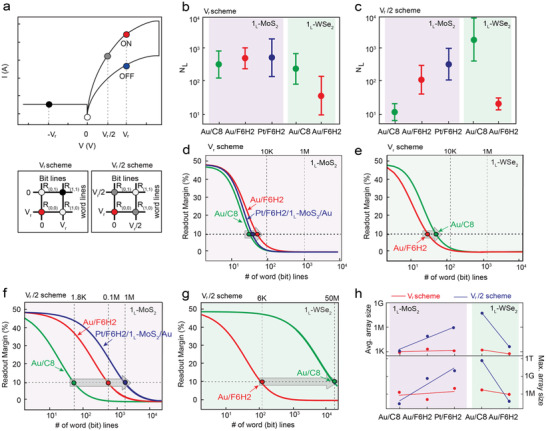
Estimated *N*_L_ and readout margin for the molecular heterojunction selectors. a) Example of conventional *I–V* switching (top) and a 2 × 2 crossbar array (bottom) for 1D‐1R in two reading voltage schemes (*V*_r_ and *V*_r_ */2* schemes). Each colored circle represents the current at the corresponding voltage (−*V*_r_, *0*, *V*_r_ */2*, and *V*_r_). The estimated *N*_L_ as a function of the molecular species (C8 and F6H2), 2D TMD (1_L_‐MoS_2_ and 1_L_‐WSe_2_), and top metal electrode (Au and Pt tip) under the b) *V*_r_ and c) *V*_r_ */2* schemes. d,e) Estimated readout margins as functions of the number of word (bit) lines and the type of 2D TMD under the *V*_r_ scheme. f,g) Estimated readout margins as functions of the number of word (bit) lines and the type of 2D TMD under the *V*_r_/2 scheme. h) Maximum size of the crossbar array as a function of the type of molecular heterojunction under the *V*_r_ scheme (linear fit in red line) and *V*_r_ */2* scheme (linear fit in blue line) based on the average *N*_L_ (top) and maximum *N*_L_ (bottom).

To examine the applicability as a molecular‐scale selector in an array structure, we investigated the *N*_L_ and readout margin for the five different molecular heterojunction systems under two different reading voltage schemes (*V*_r_ and *V*_r/_2 schemes)^[^ ^44^, ^45^ ^]^ (**Figure** **4**). In general, the crosstalk problem is one of the longstanding issues in the crossbar memory array. When the crossbar array is composed of only resistive switching memory elements, it cannot avoid generating a sneak current through unselected nodes, significantly limiting the memory capacity.^[^ ^44^, ^45^, ^46^, ^47^, ^48^ ^]^ In other words, this crosstalk problem hinders correctly encoding and reading the switching states on the designated cells, limiting the maximum size of the array.^[^ ^44^, ^45^, ^46^, ^47^, ^48^ ^]^ To resolve this issue, integration of a selector such as a transistor, a diode, or a threshold switching device element with the resistive memory at every node is commonly suggested.^[^ ^44^, ^49^, ^50^ ^]^ This integration structure can effectively reduce the sneak current through unselected cells. In addition, the cell footprint in the array can determine the memory density, so reducing the size of the selector is important. Since the molecular‐scale heterojunction could bring the ultimate dimensions to the selector itself, evaluating its applicability as the selector is worthwhile.

Figure 4a presents an example of conventional *I−V* switching characteristics for a one diode‐one resistor (1D‐1R) structure^[^ ^51^ ^]^ with two different reading voltage schemes (*V*_r_ and *V*_r/_2 schemes). The switching behavior with ON and OFF states is assumed to be exhibited only in the positive voltage region, while the current is suppressed in the negative voltage region as a whole. Note that each circle corresponds to the current at *V* = −*V*_r_, 0, *V*_r_/2, and *V*_r_. The red and blue circles represent the ON and OFF currents at *V*_r_, respectively. With these conditions, two different reading voltage schemes in the 2 × 2 crossbar form are schematized, as shown in the bottom panels of Figure 4a. Here, we assumed a “worst‐case scenario” in which the maximum sneak current where all surrounding cells are in ON states except for the selected cell is considered. Under this circumstance, depending on the reading voltage scheme, the resistance at each node can be determined (i.e., *R*_(0,0)_, *R*_(1,0)_, *R*_(0,1)_, and *R*_(1,1)_), corresponding to the colored circles in Figure 4a. Note that the selected cell corresponds to *R*_(0,0)_ and is either in the ON (red circle) or OFF (blue circle) state, while the remaining unselected cells correspond to *R*_(1,0)_, *R*_(0,1)_, and *R*_(1,1)_. Based on these resistance values, each *N*_L_ for the *V*_r_ and *V*_r_ */2* schemes can be calculated from *R*_(1,1)_/*R*_(0,0)_ and *R*_(1,0)_ /*R*_(0,0)_ or *R*_(0,1)_/*R*_(0,0)_, respectively. Here, *N*_L_ indicates the relative resistance difference between a single selected cell and other unselected cells where the applied voltage drops. Hence, a memory array with a higher *N*_L_ enables a more accurate reading of the switching state on the selected cell. Considering the *RR* and the rectification directions for the molecular heterojunctions, *V*_r_ is set to 1.5 or −1.5 V (only for the F6H2/1_L_‐WSe_2_ junction). Figure 4b,c shows the calculated *N*_L_ for the five molecular heterojunctions according to the *V*_r_ and *V*_r_ */2* schemes. Noticeably, in the case of 1_L_‐MoS_2_, the *N*_L_ in the *V*_r_ */2* scheme is largely increased from (1.3 ± 0.3) × 10^1^ to (3.7 ± 0.7) × 10^2^ when the molecular species is changed from C8 to F6H2 and the top electrode is changed from Au to Pt metal, while the *N*_L_ in the *V*_r_ scheme increases slightly (Table 1). In contrast, when 1_L_‐WSe_2_ is used, the *N*_L_ of *V*_r_ */2* is further decreased from (2.1 ± 0.4) × 10^3^ to (2.3 ± 0.3) × 10^1^ when the molecular species is changed from C8 to F6H2 compared with the *V*_r_ scheme (Table 1). Similar to *Δ*, these phenomena can be understood based on the relative difference in the transport current upon voltage application (Figure 4e; Figure S10, Supporting Information). In other words, *N*_L_ is strongly dependent on the upward (or downward) degree of band bending induced by the molecular dipole moment and metal work function and the different dominant transport pathways depending on the type of 2D TMD.

Based on the calculated *N*_L_ values for the five molecular heterojunctions, we examined the maximum size of the crossbar array (M.S.) according to a 10% readout margin based on conventional one bit‐line pull‐up (one BLPU) simulation for the *V*_r_ and *V*_r_ */2* schemes (Figure 4d–h).^[^ ^45^, ^50^, ^51^ ^]^ Note that the detailed calculation process for the readout margin is described in the Supporting Information. As shown in Figure 4d–g, the number of word (bit) lines corresponding to a 10% readout margin indicates the maximum number of lines in the crossbar array. Based on this crossing point, the maximum size of the crossbar array can be estimated. We should note that under the *V*_r_/2 scheme, the maximum size of the crossbar array can significantly increase because the sneak current through the unselected cells is further suppressed compared to the *V*_r_ scheme (Note S2, Supporting Information and Table 1). In the case of 1_L_‐MoS_2_ with the average *N*_L_, the size of the crossbar array under the *V*_r_/2 scheme can be increased from 1.8 Kbit to 1 Mbit when the molecular species is changed from C8 to F6H2 and the top electrode is changed from Au to Pt metal (Figure 4f). However, under the *V*_r_ scheme, the array size is much smaller and slightly increases from 0.9 to 1.2 Kbit (Figure 4d,h). In contrast, in the case of 1_L_‐WSe_2_ with the average *N*_L_, the array size under the *V*_r_/2 scheme decreases from 50 Mbit to 6 Kbit when the molecular species is changed from C8 to F6H2, which is a much larger reduction compared to the *V*_r_ scheme (Figure 4d,g,h). This opposite behavior is consistent with the tendency of *Δ* and *N*_L_ according to the type of 2D TMD. We also estimated the array size based on the maximum *N*_L_. 1_L_‐MoS_2_ and 1_L_‐WSe_2_ can be scaled up to 9 and 482 Gbit, respectively (Figure 4h, Table 1; Figure S11, Supporting Information). With this result, the molecular‐scale selector can be tailored by engineering the constituents of molecular heterojunctions such as the molecular dipole moments, types of 2D TMD, and contacted electrodes.

## 3 Conclusion

This work demonstrated various nonlinear rectification features in molecular heterojunctions and suggested the application potential as a tailored molecular heterojunction selector. Based on diverse interfacial band offsets achieved by changing the constituents in the molecular heterojunctions, such as the orientation of the molecular dipole moment, types of 2D TMD, and work function of the electrodes, *N*_L_ is widely tuned, scaling up to an ≈482 Gbit crossbar array. With this result, the suggested molecular heterojunction structure offers a novel route for designing a molecular‐scale selector with a high *N*_L_, which sets a good precedent for extensibility in the field of molecular electronics.

## 4 Experimental Section

4.1

4.1.1

##### Sample Preparation

The molecular SAMs of C8 and F6H2 (Sigma Aldrich) were densely packed on the Au tip (or Pt tip) by immersing the tip in the prepared molecular solutions (C8 and F6H2 with ≈5 mm 99.9% pure ethanol) for ≈2 h in a glove box filled with nitrogen gas with less than 10 ppm O_2_. The nonassembled molecules on the Au tip were removed by carefully rinsing it with ethanol droplets followed by gentle blow drying with N_2_ gas. After mechanical exfoliation of the 2D TMDs (1_L_‐MoS_2_ and 1_L_‐WSe_2_), they were carefully picked up and transferred onto each Au/SiO_2_/Si substrate by using a polydimethylsiloxane stamp coated with polypropylene carbonate. The number of 2D TMD layers was confirmed by the AFM technique (Park NX10, Park Systems Corp., South Korea) through noncontact mode. The Raman and PL spectra of the 1_L_‐2D TMDs were measured using a home‐built spectrometer equipped with a monochromator (Andor, SOLIS 303i) and a 532 nm excitation laser with a spot diameter of 0.5 µm. The signal was collected by an objective lens (100 × NA = 0.9) and dispersed by 1200 and 300 line per mm gratings for the Raman and PL measurements (Figure 1f,g; Figure S1, Supporting Information).

##### Electrical Characterization

For electrical measurement through the CAFM technique, the Au tip (or Pt tip) treated with molecular SAMs (C8 or F6H2) was carefully brought to the 1_L_‐MoS_2_ (or 1_L_‐WSe_2_)/Au/SiO_2_/Si substrate with *F*_L_ = 1 nN. The molecular heterojunction was finally formed when the Au tip with the molecular SAMs contacted the 2D TMD. The Au tip was grounded, and voltage was applied on the bottom Au electrode. The electrical *I−V* characteristics were measured in stationary mode with *F*_L_ = 1 nN using a DLPCA‐2000 built‐in current amplifier (Electro‐Optical Components) at a humidity of <15%. To statistically investigate the electrical characteristics, the Au tip was repeatedly brought to different junction points. It should be noted that, whenever the CAFM measurements were conducted, the location of the 1_L_‐MoS_2_ was confirmed based on the Raman spectra, PL, and the optical/AFM scanning images, and then the Au (or Pt) tip that was coated with the molecular SAMs was approached on that point. The current range of the *I–V* characteristics was limited in the range of around three orders of magnitude due to the limitation of the current experimental set‐up (i.e., current amplifier (DLPCA‐2000) built‐in with CAFM measurement system, Electro‐Optical Components). All the current levels at the flat region that was limited by the current measurement set‐up were excluded when investigating the electrical transport behavior. For example, the rectification ratio (*RR*), the degree of the current increase (*Δ*), the nonlinearity (*N*_L_), and the Readout Margin were investigated in relatively high voltage region.

## Conflict of Interest

The authors declare no conflict of interest.

## Supporting information

Supporting Information

## Data Availability

Research data are not shared.

## References

[1] R. L. Carroll , C. B. Gorman , Angew. Chem. 2002, 41, 4378. 12458501 10.1002/1521-3773(20021202)41:23<4378::AID-ANIE4378>3.0.CO;2-A

[2] L. A. Bumm , J. J. Arnold , M. T. Cygan , T. D. Dunbar , T. P. Burgin , L. Jones II , D. L. Allara , J. M. Tour , P. S. Weiss , Science 1996, 271, 1705.

[3] M. A. Reed , C. Zhou , C. J. Muller , T. P. Burgin , J. M. Tour , Science 1997, 278, 252.

[4] A. Nitzan , Annu. Rev. Phys. Chem. 2001, 52, 681. 11326078 10.1146/annurev.physchem.52.1.681

[5] H. Song , Y. Kim , Y. H. Jang , H. Jeong , M. A. Reed , T. Lee , Nature 2009, 462, 1039. 20033044 10.1038/nature08639

[6] R. L. McCreery , H. Yan , A. J. Bergren , Phys. Chem. Chem. Phys. 2013, 15, 1065. 23223522 10.1039/c2cp43516k

[7] L. Yuan , L. Wang , A. R. Garrigues , L. Jiang , H. V. Annadata , M. A. Antonana , E. Barco , C. A. Nijhuis , Nat. Nanotechnol. 2018, 13, 322. 29581549 10.1038/s41565-018-0068-4

[8] J. Shin , K. Gu , S. Yang , C.‐H. Lee , T. Lee , Y. H. Jang , G. Wang , Nano Lett. 2018, 18, 4322. 29906125 10.1021/acs.nanolett.8b01294

[9] L. Yuan , N. Nerngchamnong , L. Cao , H. Hamoudi , E. Del Barco , M. Roemer , R. K. Sriramula , D. Thompson , C. A. Nijhuis , Nat. Commun. 2015, 6, 6324. 25727708 10.1038/ncomms7324

[10] C. Fang , P. Zhao , B. Cui , L. Wang , D. Liu , S. Xie , Phys. Lett. A 2010, 374, 4465.

[11] C. A. Nijhuis , W. F. Reus , G. M. Whitesides , J. Am. Chem. Soc. 2010, 132, 18386. 21126089 10.1021/ja108311j

[12] M. Alemani , M. V. Peters , S. Hecht , K.‐H. Rieder , F. Moresco , L. Grill , J. Am. Chem. Soc. 2006, 128, 14446. 17090013 10.1021/ja065449s

[13] M. Irie , S. Kobatake , M. Horichi , Science 2001, 291, 1769. 11230689 10.1126/science.291.5509.1769

[14] Y. Han , C. Nickle , Z. Zhang , H. P. A. G. Astier , T. J. Duffin , D. Qi , Z. Wang , E. Del Barco , D. Thompson , C. A. Nijhuis , Nat. Mater. 2020, 19, 843. 32483243 10.1038/s41563-020-0697-5

[15] T. Inoshita , S. Saito , H. Hosono , Small Sci. 2021, 1, 2100020. 10.1002/smsc.202100020 PMC11935841 40213406

[16] A. Vezzoli , R. J. Brooke , S. J. Higgins , W. Schwarzacher , R. J. Nichols , Nano Lett. 2017, 17, 6702. 28985083 10.1021/acs.nanolett.7b02762

[17] E. Margapoti , J. Li , Ö. Ceylan , M. Seifert , F. Nisic , T. L. Anh , F. Meggendorfer , C. Dragonetti , C. A. Palma , J. V. Barth , J. J. Finley , Adv. Mater. 2015, 27, 1426. 25641369 10.1002/adma.201405110

[18] J. Shin , S. Yang , Y. Jang , J. S. Eo , T.‐W. Kim , T. Lee , C.‐H. Lee , G. Wang , Nat. Commun. 2020, 11, 1. 32179744 10.1038/s41467-020-15144-9 PMC7075907

[19] K. Demirkan , A. Mathew , C. Weiland , Y. Yao , A. Rawlett , J. M. Tour , R. L. Opila , J. Chem. Phys. 2008, 128, 074705. 18298162 10.1063/1.2832306

[20] W. E. Ford , D. Gao , N. Knorr , R. Wirtz , F. Scholz , Z. Karipidou , K. Ogasawara , S. Rosselli , V. Rodin , G. Nelles , F. Von Wronchem , ACS Nano 2014, 8, 9173. 25093963 10.1021/nn502794z

[21] V. Diez‐Cabanes , D. C. Morales , M. Souto , M. Paradinas , F. Delchiaro , A. Painelli , C. Ocal , D. Cornil , J. Cornil , J. Veciana , I. Ratera , Adv. Mater. Technol. 2019, 4, 1800152.

[22] P. C. Rusu , G. Brocks , J. Phys. Chem. B 2006, 110, 22628. 17092010 10.1021/jp0642847

[23] C. Huang , H. E. Katz , J. E. West , Langmuir 2007, 23, 13223. 18020470 10.1021/la702409m

[24] M. Salinas , C. M. Jäger , A. Y. Amin , P. O. Dral , T. Meyer‐Friedrichsen , A. Hirsch , T. Clark , M. Halik , J. Am. Chem. Soc. 2012, 134, 12648. 22731755 10.1021/ja303807u

[25] B. Yuan , P. Chen , J. Zhang , Z. Cheng , X. Qiu , C. Wang , Chin. Sci. Bull. 2013, 58, 3630.

[26] G. Wang , H. Jeong , J. Ku , S.‐I. Na , H. Kang , E. Ito , Y. H. Jang , J. Noh , T. Lee , J. Colloid Interface Sci. 2014, 419, 39. 24491327 10.1016/j.jcis.2013.12.052

[27] M. Baghbanzadeh , L. Belding , L. Yuan , J. Park , M. H. Al‐Sayah , C. M. Bowers , G. M. Whitesides , J. Am. Chem. Soc. 2019, 141, 8969. 31072101 10.1021/jacs.9b02891

[28] A. Kovalchuk , D. A. Egger , T. Abu‐Husein , E. Zojer , A. Terfort , R. C. Chiechi , RSC Adv. 2016, 6, 69479. 10.1039/c5sc03097h PMC5953005 29896361

[29] B. de Boer , A. Hadipour , M. M. Mandoc , T. van Woudenbergh , P. W. Blom , Adv. Mater. 2005, 17, 621.

[30] D. M. Alloway , M. Hofmann , D. L. Smith , N. E. Gruhn , A. L. Graham , R. Colorado Jr , V. H. Wysocki , T. R. Lee , P. A. Lee , N. R. Armstrong , J. Phys. Chem. B 2003, 107, 11690.

[31] O. Zenasni , A. C. Jamison , T. R. Lee , Soft Matter 2013, 9, 6356.

[32] A. Vilan , D. Cahen , Chem. Rev. 2017, 117, 4624. 28230354 10.1021/acs.chemrev.6b00746

[33] L. Xie , Nanoscale 2015, 7, 18392. 26508084 10.1039/c5nr05712d

[34] P. Tonndorf , R. Schmidt , P. Böttger , X. Zhang , J. Börner , A. Liebig , M. Albrecht , C. Kloc , O. Gordan , D. R. Zahn , S. M. De Vasconcellos , R. Bratschitsch , Opt. Express 2013, 21, 4908. 23482024 10.1364/OE.21.004908

[35] X. Zhang , X.‐F. Qiao , W. Shi , J.‐B. Wu , D.‐S. Jiang , P.‐H. Tan , Chem. Soc. Rev. 2015, 44, 2757. 25679474 10.1039/c4cs00282b

[36] K.‐C. Liao , C. M. Bowers , H. J. Yoon , G. M. Whitesides , J. Am. Chem. Soc. 2015, 137, 3852. 25751593 10.1021/jacs.5b00137

[37] C. Naud , P. Calas , A. Commeyras , Langmuir 2001, 17, 4851.

[38] K. Tamada , T. Ishida , W. Knoll , H. Fukushima , R. Colorado Jr , M. Graupe , O. E. Shmakova , T. R. Lee , Langmuir 2001, 17, 1913.

[39] S. Kobayashi , T. Nishikawa , T. Takenobu , S. Mori , T. Shimoda , T. Mitani , H. Shimotani , N. Yoshimoto , S. Ogawa , Y. Iwasa , Nat. Mater. 2004, 3, 317. 15064756 10.1038/nmat1105

[40] A. Allain , A. Kis , ACS Nano 2014, 8, 7180. 24949529 10.1021/nn5021538

[41] Y. Guo , J. Robertson , Microelectron. Eng. 2015, 147, 184.

[42] D. Çakır , C. Sevik , F. M. Peeters , J. Mater. Chem. C 2014, 2, 9842.

[43] S. Kim , Y.‐F. Chang , B.‐G. Park , RSC Adv. 2017, 7, 17882.

[44] G. Wang , A. C. Lauchner , J. Lin , D. Natelson , K. V. Palem , J. M. Tour , Adv. Mater. 2013, 25, 4789. 23836363 10.1002/adma.201302047

[45] G. Wang , J.‐H. Lee , Y. Yang , G. Ruan , N. D. Kim , Y. Ji , J. M. Tour , Nano Lett. 2015, 15, 6009. 26252444 10.1021/acs.nanolett.5b02190

[46] M.‐J. Lee , S. Seo , D.‐C. Kim , S.‐E. Ahn , D. H. Seo , I.‐K. Yoo , I.‐G. Baek , D.‐S. Kim , I.‐S. Byun , S.‐H. Kim , I.‐R. Hwang , J.‐S. Kim , S.‐H. Jeon , B.‐H. Park , Adv. Mater. 2007, 19, 73.

[47] E. Linn , R. Rosezin , C. Kügeler , R. Waser , Nat. Mater. 2010, 9, 403. 20400954 10.1038/nmat2748

[48] J. W. Seo , S. J. Baik , S. J. Kang , Y. H. Hong , J. H. Yang , K. S. Lim , Appl. Phys. Lett. 2011, 98, 233505.

[49] R. Aluguri , D. Kumar , F. M. Simanjuntak , T.‐Y. Tseng , AIP Adv. 2017, 7, 095118.

[50] F. Gül , Results Phys. 2019, 12, 1091.

[51] J.‐J. Huang , Y.‐M. Tseng , C.‐W. Hsu , T.‐H. Hou , IEEE Electron Device Lett. 2011, 32, 1427.

