# Supplementary material for: Tailoring the Interfacial Band Offset by the Molecular Dipole Orientation for a Molecular Heterojunction Selector
Source: Adv Sci (Weinh). 2021 Sep 9;8(21):2101390. doi: 10.1002/advs.202101390 (PMC8564428; doi:10.1002/advs.202101390)
Supplement: Supplementary file 1 — Supporting Information [file ADVS-8-2101390-s001.pdf]

© 2021 Wiley-VCH GmbH

ADVANCED SCIENCE
Open Access

Supporting Information

for *Adv. Sci.*, DOI: 10.1002/advs.202101390

Tailoring the interfacial band offset by the molecular dipole orientation for a molecular heterojunction selector

*Jung Sun Eo, Jaeho Shin, Seunghoon Yang, Takgyeong Jeon, Jaeho Lee, Sanghyeon Choi, Chul-Ho Lee and Gunuk Wang**

Supporting Information

Tailoring the interfacial band offset by the molecular dipole orientation for a molecular heterojunction selector

Jung Sun Eo†, Jaeho Shin†, Seunghoon Yang, Takgyeong Jeon, Jaeho Lee, Sanghyeon Choi, Chul-Ho Lee and Gunuk Wang*

†These authors contributed equally to this work.

J. S. Eo, J. Shin, S. Choi, T. Jeon, S. Yang, J. Lee, Prof. C.-H. Lee and Prof. G. Wang
KU-KIST Graduate School of Converging Science and Technology, Korea University, 145, Anam-ro, Seongbuk-gu, Seoul 02841, Republic of Korea

Prof. C.-H. Lee and Prof. G. Wang
Department of Integrative Energy Engineering, Korea University, 145, Anam-ro, Seongbuk-gu, Seoul 02841, Republic of Korea

E-mail: gunukwang@korea.ac.kr

Keywords: Molecular electronics, Molecular heterojunction, Two-dimensional semiconductor, Molecular dipole moment, and Molecular selector

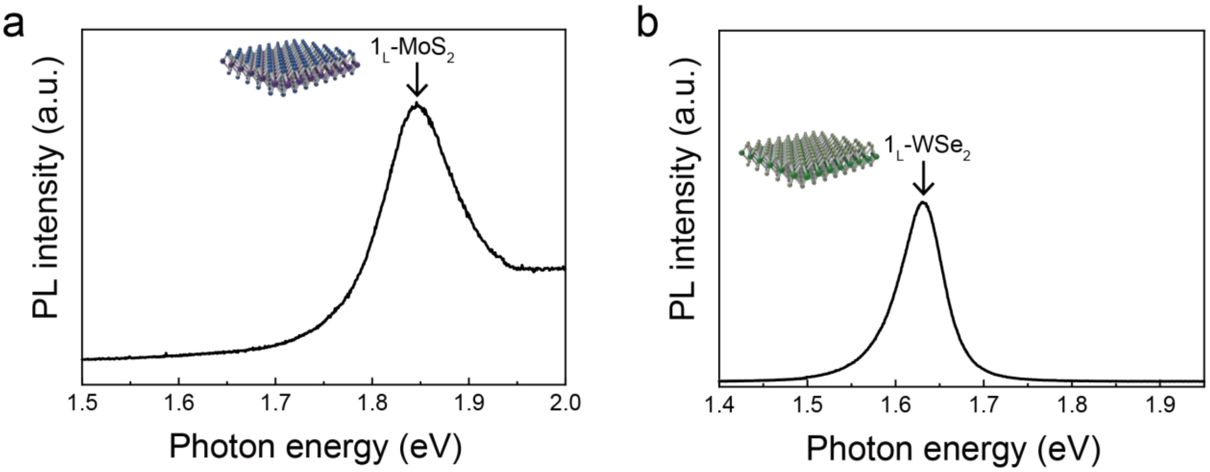

Figure S1. Characterization of 1_L-2D TMDs. PL spectra of (**a**) 1_L-MoS_2 and (**b**) 1_L-WSe_2, where the arrows represent the strong excitonic emission peaks.

Supporting Information Note 1

- Hertzian elastic contact model and effective molecular dipole moments

The Hertzian elastic contact model[1] can be utilized to estimate the contact radius ($R_{cont.}$) between Au or Pt tip and molecular SAMs, $R_{cont.} = (R_{tip}P_n/K)^{1/3}$, where R_{tip} is the Au or Pt-tip radius of ~34 nm and ~24 nm, respectively (Fig. S2), P_n is the net force determined by F_L (tip-loading force) + $F_{adhesion}$ (adhesion force), and K is elastic modulation (~20 GPa)[2]. Note that it was found as follows that $F_{adhesion}$ = 18.99 nN, 6.69 nN, and 6.41 nN for Au/C8, Au/F6H2, and Pt/F6H2, respectively (Fig. S3). Based on this model, we estimated the $R_{cont.}$ for all molecular heterojunctions, as summarized in Table S1.

Based on the R_{tip} and $R_{cont.}$ for the molecular heterojunctions, we estimated contained angle (θ) = $2R_{cont.}/R_{tip} \times 360/2\pi$, exhibiting from 7.96 to 10.93°. Therefore, the vertical component of the molecular dipole moment is changed from $\mu_\perp$ to $\mu_\perp \times \cos(\theta/2)$ (Fig. S4). The value of $\cos(\theta/2)$ is varied 0.995 to 0.998 according to the types of molecular heterojunctions (Table S1). Therefore, although the contact curvature of the tip (tip geometry) can affect the direction/magnitude of the molecular "effective" dipole moments, its effect seems to be negligible.

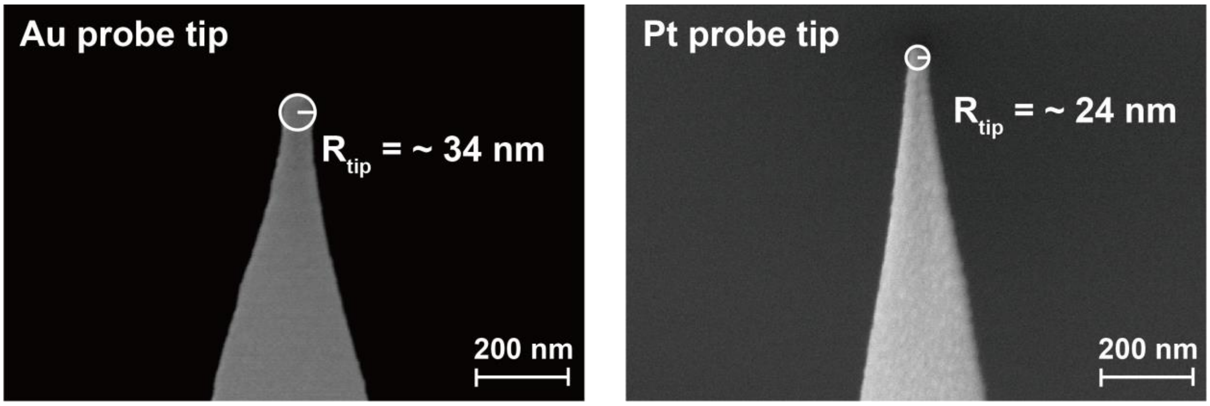

Figure S2. SEM images of the Au (left) and Pt (right) probe tips for the CAFM technique.

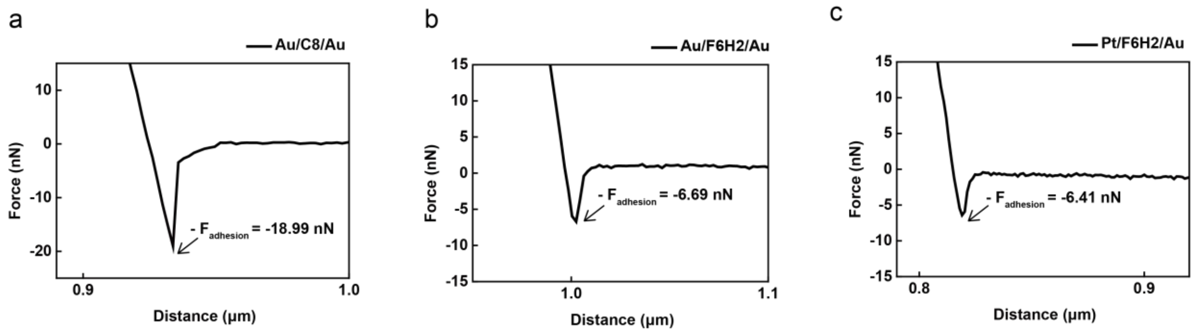

Figure S3. Representative Force-Distance (*F-D*) curves for the (**a**) Au/C8/Au, (**b**) Au/F6H2/Au, and (**c**) Pt/F6H2/Au junctions, respectively. The local minimum indicates the adhesion force ($F_{adhesion}$) which was found as follows that $F_{adhesion}$ = 18.99 nN, 6.69 nN, and 6.41 nN for Au/C8, Au/F6H2, and Pt/F6H2, respectively.

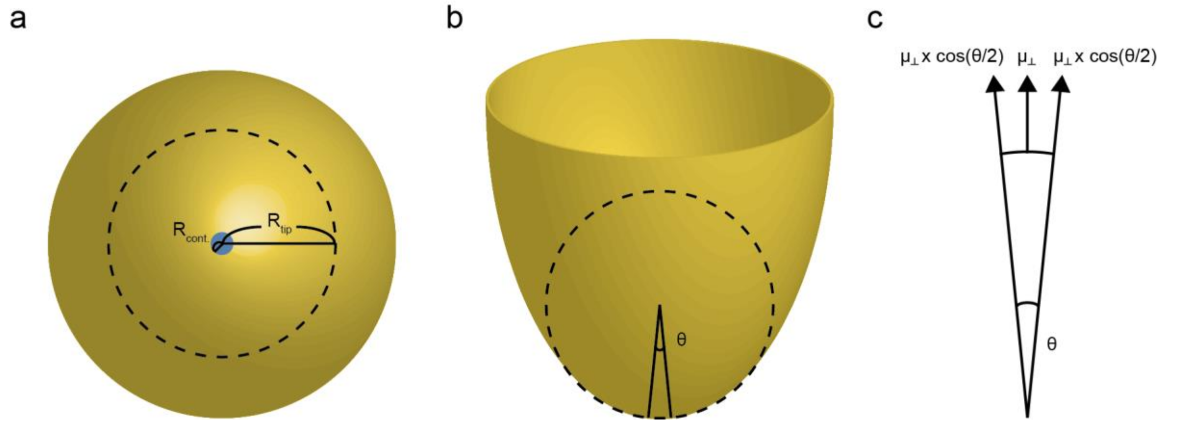

Figure S4. (**a**) Bottom view schematic of AFM tip. The blue colored region is the effective contact area. (**b**) The front view schematic of AFM tip, indicating the contained angle (θ) of the effective contact area. (**c**) The vertical component of molecular dipole moment depending on effective contact curvature of the tip.

**Molecular Heterojunction**	***R_{tip}* (nm)**	***P_n* (nN)**	***$R_{cont.}$* (nm)**	**θ (°)**	**cos (θ/2)**
**Au/C8/(1_L-MoS_2 or 1_L-WSe_2/Au**	34	19.995	3.24	10.93	0.995
**Au/F6H2/(1_L-MoS_2 or 1_L-WSe_2/Au**	34	7.690	2.36	7.96	0.998
**Pt/F6H2/1_L-MoS_2/Au**	24	7.415	2.07	9.89	0.996

Table S1. Summarization of R_{tip}, P_n, $R_{cont.}$, θ, and cos(θ/2) for all of our molecular heterojunctions.

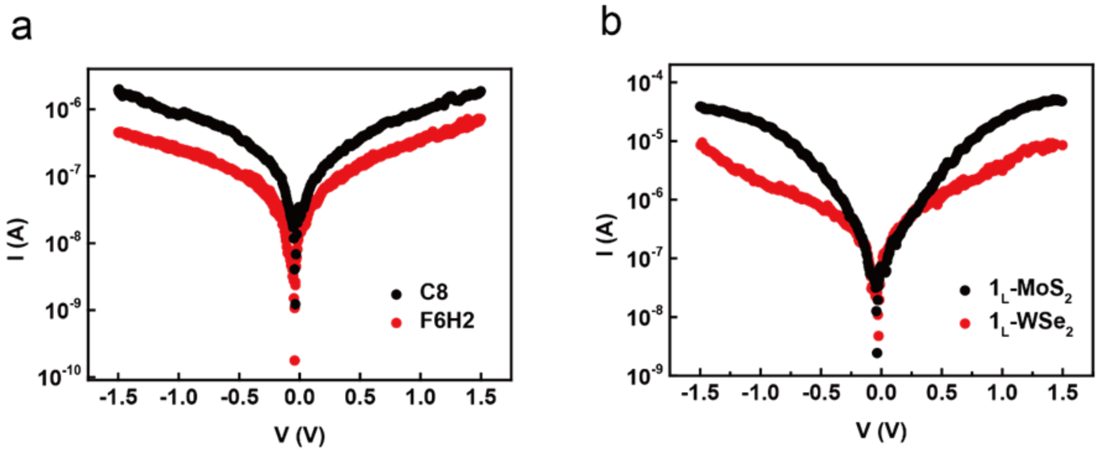

Figure S5. Representative *I*-*V* characteristics of the (**a**) Au/C8/Au (black), Au/F6H2/Au (red), (**b**) Au/1_L-MoS_2/Au (black), and Au/1_L-WSe_2/Au (red) junctions, respectively.

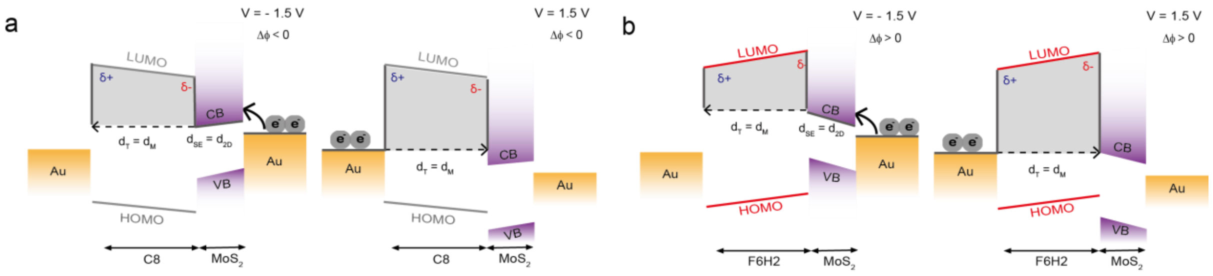

Figure S6. Schematics of interfacial energy band offsets for the molecular heterojunction of (**a**) Au/C8/1_L-MoS_2/Au and (**b**) Au/F6H2/1_L-MoS_2/Au when V = –1.5 V (left) or V= 1.5 V (right) is applied, respectively. When V = 1.5 V, the CB edge of 1_L-MoS_2 can be located in the bias window in both molecular heterojunctions regardless of the molecular species or the bending degree, resulting in a higher transport current than when V = -1.5 V.

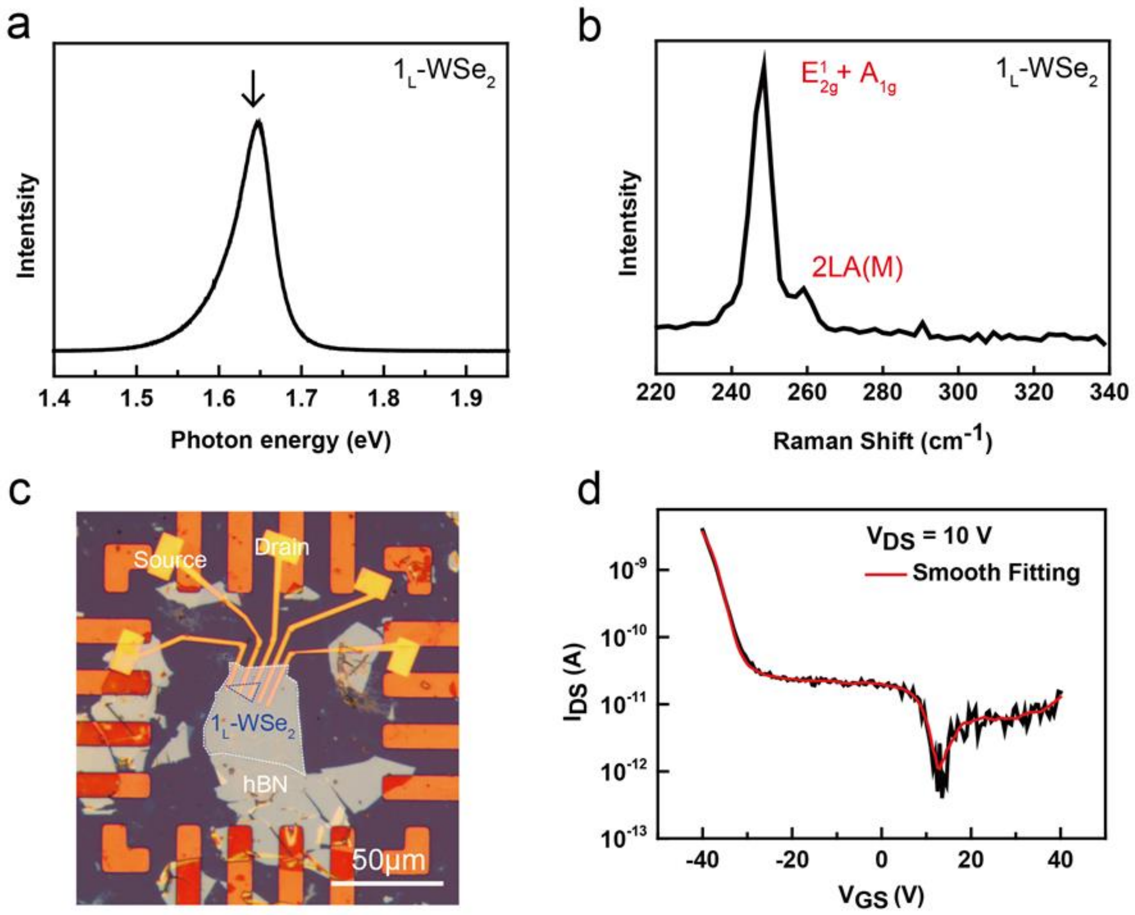

Figure S7. (**a**) PL spectra and (**b**) Raman spectra of the 1_L-WSe_2 flake on h-BN. (**c**) The optical image of 1_L-WSe_2 FET device. (**d**) The transfer characteristic curve (I_{DS} vs V_{GS}) for the 1_L-WSe_2 FET device with Au contacts, showing typical *p*-type carrier transport. Red line represents the smooth line fitting.

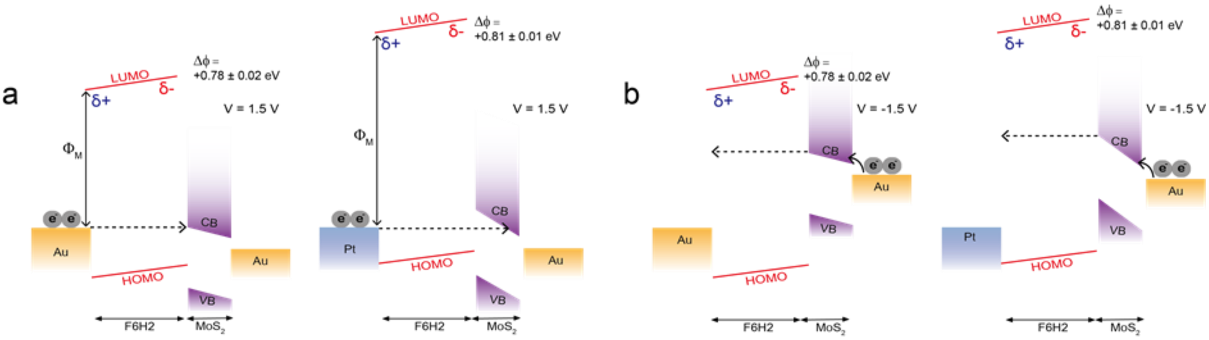

Figure S8. Schematics of interfacial energy band offsets for the Metal-tip (Au or Pt)/F6H2/1_L-MoS_2/Au molecular heterojunction when (**a**) V = 1.5 V and (**b**) V = -1.5 V is applied.

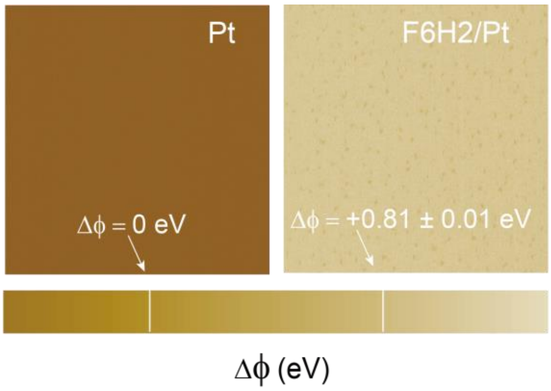

Figure S9. SKPM images of bare Pt (left) and F6H2 on Pt (right). $\Delta\phi = 0.81 \pm 0.01$, which is similar to that of F6H2 on Au.

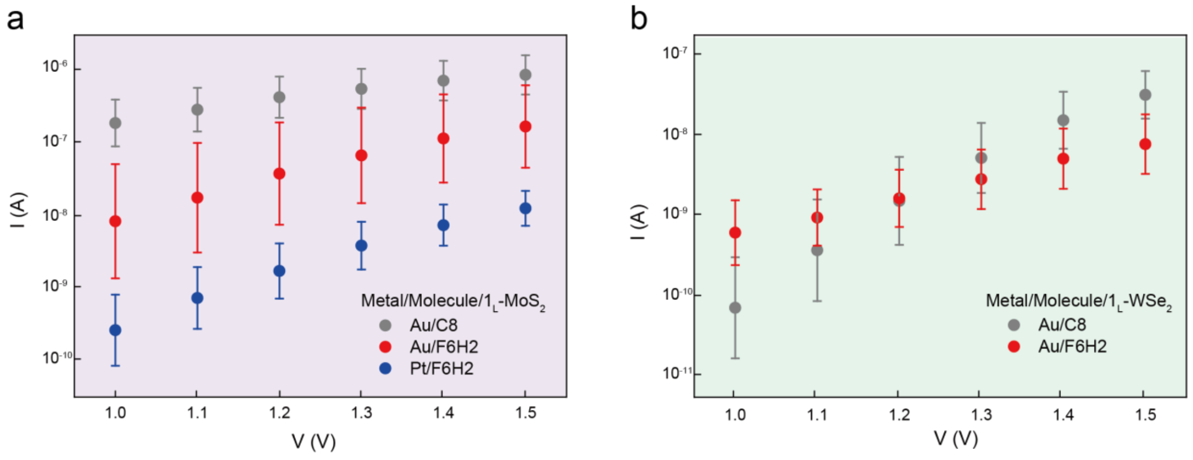

Figure S10. Plots of transport currents for the molecular heterojunctions with different 2D TMDs ((**a**) 1_L-MoS_2 and (**b**) 1_L-WSe_2) when the applied voltage is increased from 1.0 to 1.5 V.

Supporting Information Note 2

- Calculation method of one bit-line pull-up (one BLPU)

To estimate the maximum size of the crossbar array ($N \times N$), the readout margin was calculated via one bit-line pull-up (one BLPU) simulation considering "the worst case scenario". Based on the equivalent circuit of the one BLPU scheme, the corresponding Kirchhoff equation is used to obtain the readout margin.[3-5] In general, the 10% readout margin (pull-up voltage ($\Delta V/V_{pu}$)) has been used as the minimum criterion to differentiate the switching states.[3-6] The Kirchhoff equation that provides the number of word/bit lines (N) via normalized V_{pu} is as follows:

$$\frac{\Delta V}{V_{pu}} = \frac{\mathrm{R}_{pu}}{\left[R^F_{on} \,||\, \left(\frac{2R^F_{sneak}}{(N-1)}\right)+\left(\frac{R^R_{sneak}}{(N-1)^2}\right)\right]+\mathrm{R}_{pu}} - \frac{\mathrm{R}_{pu}}{\left[R^F_{off} \,||\, \left(\frac{2R^F_{sneak}}{(N-1)}\right)+\left(\frac{R^R_{sneak}}{(N-1)^2}\right)\right]+\mathrm{R}_{pu}} \quad .$$

The calculated readout margin dependent on the voltage drop (ΔV) across the pull-up resistor (R_{pu}) reflects how correctly the switching state on the selected cell is read. We assumed unipolar switching memory, where its *I-V* behavior in the ON state is precisely the same as that of molecular heterojunction selectors. Since the "worst-case scenario" is considered, all the unselected cells are designated as having the resistance in the ON state. This gives the R^F_{sneak} and R^R_{sneak} values determined via the experimental *I-V* curves of the molecular heterojunction selector. R^R_{sneak} ($R_{(1,1)}$) and R^F_{sneak} ($R_{(0,1)}$ and $R_{(1,0)}$) are designated for the unselected cells (Fig. 4a). R^F_{on} ($R_{(0,0)}$ in the ON state) and R^F_{off} ($R_{(0,0)}$ in the OFF state) are designated for the selected cells (Fig. 4a). In our case, R^F_{on} is assumed to be the resistance at V_r = 1.5 V or V_r = –1.5 V (only for F6H2/1_L-WSe_2), and R^F_{off} is assumed to be the resistance at V_r = 0.5 V or V_r = –0.5 V (only for F6H2/1_L-WSe_2), where the ON-OFF ratio is set modestly.

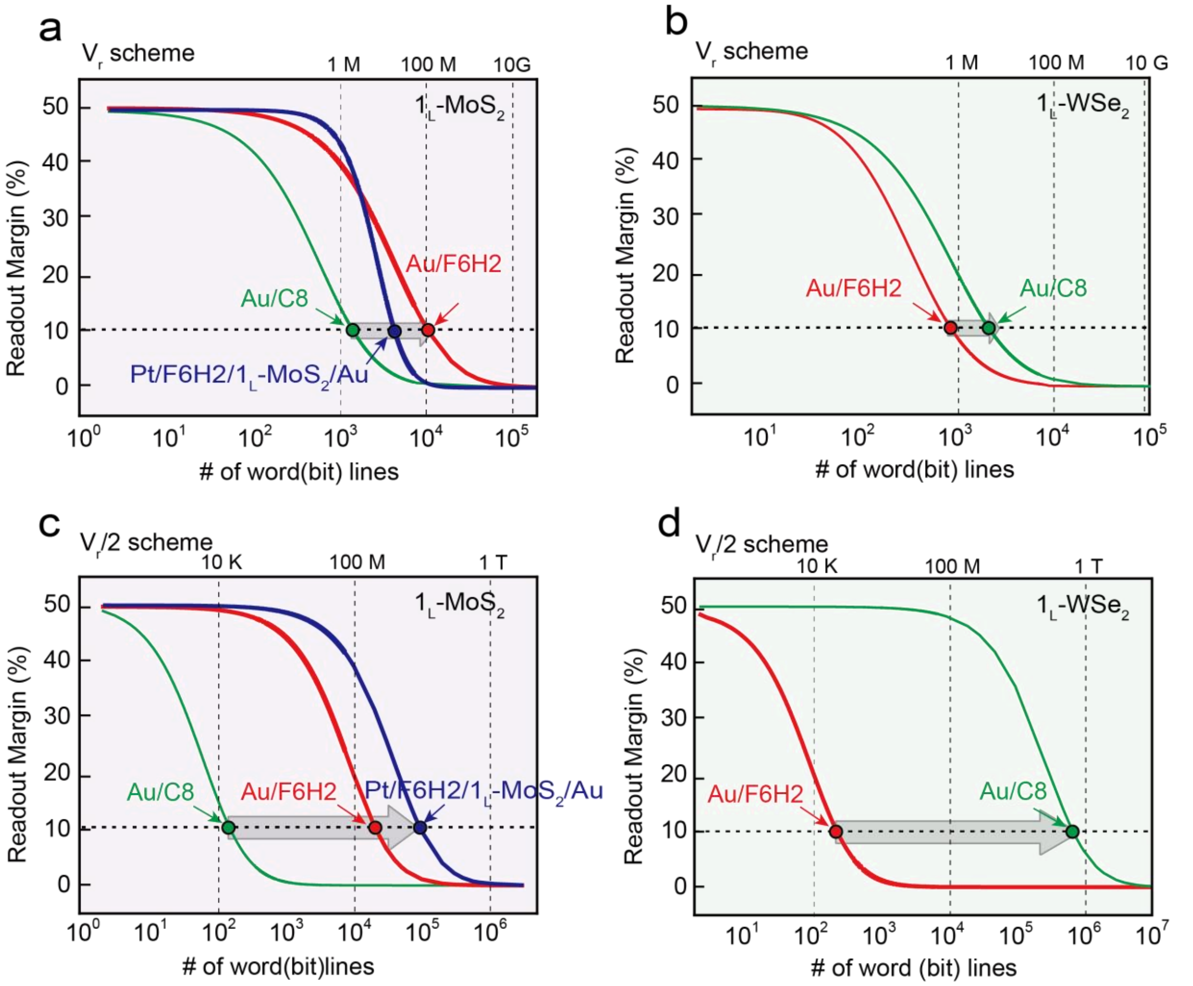

Figure S11. (a, b) Estimated readout margins as functions of the number of word (bit) lines and the type of 2D TMD under the V_r scheme. **(c, d)** Estimated readout margins as functions of the number of word (bit) lines and the type of 2D TMD under the $V_r/2$ scheme. Note that the maximum N_L values for all molecular heterojunctions are assumed.

[1] K. L. Johnson *Cambridge University Press:* New York, **1985**, 104.
[2] H. Song, H. Lee, T. Lee *J. Am. Chem. Soc.* **2007**, *129*, 3806.
[3] C.-L. Lo, T.-H. Hou, M.-C. Chen, J.-J. Huang, *IEEE Trans. Electron Devices* **2012**, *60*, 420.
[4] G. Wang, J.-H. Lee, Y. Yang, G. Ruan, N. D. Kim, Y. Ji, J. M. Tour, *Nano Lett.* **2015**, *15*, 6009.
[5] F. Gül, *Results Phys.* **2019**, *12*, 1091.
[6] J.-J. Huang, Y.-M. Tseng, C.-W. Hsu, T.-H. Hou, *IEEE Electron Device Lett.* **2011**, *32*, 1427.
